# Biomarkers in sepsis: a systematic review

**DOI:** 10.1186/cc14130

**Published:** 2015-03-16

**Authors:** F Morriello, J Marshall

**Affiliations:** 1 Institute of Medical Sciences, Toronto, ON, Canada; 2 St. Michael's Hospital, Toronto, ON, Canada

## Introduction

Sepsis is a common reason for admission to ICUs throughout the world. During the past two decades, the incidence of sepsis in the USA has tripled and is now the 10th leading cause of death. As sepsis continues to impact negatively on critically ill patients, it is clear that early diagnosis and effective management could improve patient morbidity and mortality. Numerous studies have attempted to examine biomarkers and their ability to diagnose and prognosticate septic patients. Despite multiple efforts, currently there are no reliable markers that can effectively improve our clinical effectiveness in diagnosing and managing septic patients. The purpose of our systematic review was to evaluate the diagnostic and prognostic value of various biomarkers used in septic patients.

## Methods

A systematic search of the literature was performed with MEDLINE, EMBASE, and the Cochrane Central Register of Controlled Trials databases using terminology selected for biomarkers (through to and including November 2013). All articles involving neonates and not in English were excluded. Inclusion was agreed on by two independent reviewers of abstracts or full text. Assessment was based on the biomarker's ability to diagnose septic patients and its ability to predict mortality.

## Results

Of 5,257 articles identified, all abstracts were screened, and 750 full-text articles were selected for review. These included primarily randomized controlled trials, cohort studies and postmortem studies. Of 49 biomarkers examined, 72% of the studies examined procalcitonin. Comparing the serum of septic patients with that of controls, most biomarkers were elevated in septic patients, even though only a few had high sensitivity (>85%) and high specificity (>80%). It was often difficult to compare study group with control group as the control group patients were usually not healthy controls.

## Conclusion

Overall the heterogeneity of studies, small sample size and the lack of true healthy controls influenced the ability to use the biomarker for prognostication of a septic patient. Furthermore, the lack of healthy control raises the question of redefining selection criteria in order to better study septic patients.

